# Performance Parameters and Characterizations of Nanocrystals: A Brief Review

**DOI:** 10.3390/pharmaceutics8030026

**Published:** 2016-08-30

**Authors:** Manasi M. Chogale, Vinod N. Ghodake, Vandana B. Patravale

**Affiliations:** Department of Pharmaceutical Sciences and Technology, Institute of Chemical Technology, Nathalal Parekh Marg, Matunga, Mumbai-400019, India; manasi4190@gmail.com (M.M.C.); g.vinod8863@gmail.com (V.N.G.)

**Keywords:** nanocrystals, dissolution velocity, saturation solubility, thermal analysis, X-ray diffraction, FT-IR, Raman spectroscopy, particle size, surface morphology, particle shape

## Abstract

Poor bioavailability of drugs associated with their poor solubility limits the clinical effectiveness of almost 40% of the newly discovered drug moieties. Low solubility, coupled with a high log *p* value, high melting point and high dose necessitates exploration of alternative formulation strategies for such drugs. One such novel approach is formulation of the drugs as “Nanocrystals”. Nanocrystals are primarily comprised of drug and surfactants/stabilizers and are manufactured by “top-down” or “bottom-up” methods. Nanocrystals aid the clinical efficacy of drugs by various means such as enhancement of bioavailability, lowering of dose requirement, and facilitating sustained release of the drug. This effect is dependent on the various characteristics of nanocrystals (particle size, saturation solubility, dissolution velocity), which have an impact on the improved performance of the nanocrystals. Various sophisticated techniques have been developed to evaluate these characteristics. This article describes in detail the various characterization techniques along with a brief review of the significance of the various parameters on the performance of nanocrystals.

## 1. Introduction

The most tedious challenge currently faced by formulation scientists is that of formulation of poorly water-soluble drug moieties. Advancement in high throughput screening methods is mainly responsible to an even larger number of newly discovered drugs having poor water solubility. According to literature reports, more than 40% of the drugs being introduced into the formulation research pipeline have poor water solubility [1,2]. Poor water solubility of drugs translates into poor bioavailability, thereby affecting the clinical efficacy of these drugs. Strategies to improve the water solubility of these drugs are being explored in an attempt to improve their bioavailability.

Many approaches have been researched in this endeavour of bioavailability enhancement. However, most of these methods are constrained to drugs possessing a specific chemistry (i.e., solubility in certain organic solvents) or with a definite molecular size/shape or conformation (i.e., for molecules to be incorporated into cyclodextrin ring structure) [3]. Moreover, the use of surfactants also provides a viable alternative, albeit the toxicity with the use of surfactants may present an issue. Development of the drug into nanosized formulations have helped overcome the bioavailability problems of many a poorly water soluble drugs belonging to BCS Class 2 and 4. One category of such nanosized formulations comprise of nanoscaled drug particles stabilized by a suitable stabilizer or surfactant, referred to as “Nanocrystals”. Drug nanocrystals are defined as crystals with a size in the nanometer range; usually ranging from a few nanometers to 1000 nm. Drug nanocrystals when dispersed in an aqueous medium are referred to as “Nanosuspensions”. An additional characteristic of the nanocrystals is that it is composed of 100% drug, devoid of any carrier component as in polymeric/lipidic nanoparticles. They are usually prepared either by precipitation of the drug from its organic solvent following addition of an aqueous surfactant/stabilizers solution or by subjecting the macrosized dispersion of the drug to intensive particle size reduction methods in presence of a surfactant/stabilizer.

## 2. Performance Attributes of Nanocrystals

Nanocrystals are bestowed with the ability to enhance the dissolution velocity and bioavailability of BCS Class 2 and 4 drugs owing to their very specific properties. The properties of nanocrystals responsible for their enhanced performance are enlisted and briefly discussed in the following sections.

### 2.1. Enhancement of Dissolution Velocity by Surface Area Enlargement

The size reduction of a drug leads to an improved surface area and thus according to the Noyes-Whitney equation to an enhanced dissolution velocity [4]. Therefore, for drugs with dissolution velocity as the rate limiting step, micronization maybe a suitable approach to successfully augment the bioavailability. By progressing from micronization further down to nanonization, the particle surface and thereby the dissolution velocity is further enhanced.

### 2.2. Increase in Saturation Solubility

**Figure 1:**
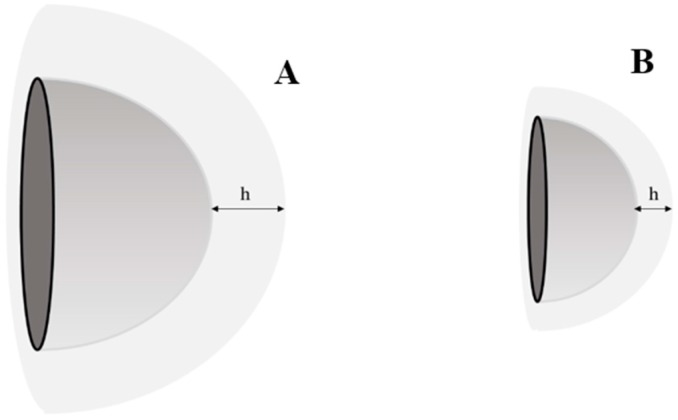
Comparison of Diffusional Distance of a (**A**) Microcrystal and a (**B**) Nanocrystal.

The universal statement is that the saturation solubility is a constant value and function of the compound, the dissolution medium and the temperature conditions. This concept is relevant for powders with size in the micrometer range or more. However, below a critical size of 1–2 μm, the saturation solubility is also dependant on the crystalline structure and particle size. It increases with a reduction in particle size below 1000 nm. The enhancement of dissolution velocity of nanocrystals can be explained by the Noyes–Whitney equation [4,5]. For drug nanocrystals, the enhancement in saturation solubility (*C*) and surface area (*A*) leads to a rise in the dissolution velocity (*dX*/*dt*).

(1) $$\frac{dX}{dt}=\;\frac{DA}{h}*\left(Cs-Ct\right)$$

where *dX*/*dt* is the dissolution velocity, *D* is the diffusion coefficient, *A* is the surface area, *h* is the diffusional distance, *Cs* is the saturation solubility, and *Ct* is the concentration around the particles (Refer Figure 1). An imperative factor herein is the diffusional distance “*h*”, which as a part of the hydrodynamic boundary layer *h_H_*, is also strongly reliant on the particle size, as the Prandtl equation shows [6]:

*h_H_* = *k* (*L*^1/2^/*V*^1/2^) (2)

where *h_H_* is the hydrodynamic boundary layer, *k* denotes a constant, *L* is the length of the particle surface, and *V* is the relative velocity of the flowing liquid surrounding the particle.

According to the Prandtl equation, decrease in particle size leads to a reduced diffusional distance h and subsequently an enhanced dissolution velocity, as described by the Noyes–Whitney equation. Due to the enhanced saturation solubility, the concentration gradient between gut lumen and blood augments and accordingly absorption by passive diffusion rises. Hence to conclude, a decrease in the particle size of the drug to the nanometer range leads to an enhancement in solubility and consequently the dissolution velocity. Both solubility and dissolution velocity are very critical factors in terms of improvement of the bioavailability of poorly soluble drugs.

Broadly speaking, solubility is best for the polymorphic form that is identified by maximum energy and minimum melting point. Amorphous portion generated in homogenization progress also imparts an enhanced solubility due to enhanced inner energy of substance in this state [7]. Dependence of saturation solubility on the particle size can be explained by the Kelvin and the Ostwald–Freundlich equations [6,8].

The Kelvin equation was ideally used to describe the vapor pressure over a curved surface of a liquid droplet in gas; it is also appropriate to explain the relation between the dissolution pressure and the curvature of the solid particles in liquid:

(3) $$Ln\;\frac{Pr}{P\infty }=\frac{2\gamma Mr}{rRT\rho }$$

where *Pr* is the dissolution pressure of a particle with the radius *r*, *P*∞ is the dissolution pressure of an infinitely large particle, *γ* is the surface tension, *R* is the gas constant, *T* is the absolute temperature, *r* is the radius of the particle, *Mr* is the molecular weight, and ρ is the density of the particle.

**Figure 2:**
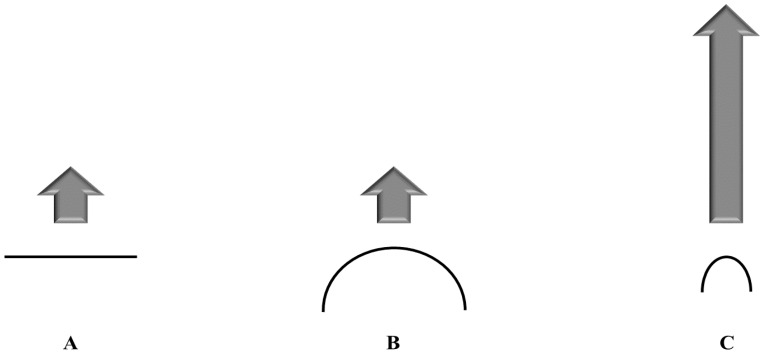
Increasing Dissolution Pressure Over (**A**) a Flat Surface; (**B**) Curvature of a Microparticle; (**C**) Curvature of a Nanoparticle.

According to the Kelvin equation, the dissolution pressure increases with enhanced curvature, which means reduced particle size. (Refer Figure 2).

Particles in the nanometer range offer a huge curvature; consequently a higher dissolution pressure is achieved driving the equilibrium towards dissolution. The Ostwald–Freundlich equation correlates saturation solubility of the drug and the particle size:

(4) $$\log\frac{Cs}{C\alpha }=\frac{2ᴩV}{2.303RTᴩr}$$

where *Cs* is the saturation solubility, *Cα* is the solubility of the solid consisting of large particles, *r* is the interfacial tension of substance, *V* is the molar volume of the particle material, *R* is the gas constant, *T* is the absolute temperature, ᴩ is the density of the solid, and *r* is the radius.

It is apparent that the saturation solubility (*Cs*) of the drug increases with a decrease in particle size (*r*). However, this effect is more pronounced for materials that have mean particle size of less than 2 μm.

### 2.3. Increased Adhesiveness

Another ideal feature of nanocrystals is a marked enhancement in adhesiveness compared to microparticles. The increased adhesiveness, a general characteristic of nanoparticles, is responsible for improving the oral absorption of poorly soluble drugs besides the increased saturation solubility and dissolution velocity [9]. Mucoadhesive (adhesion to mucous surfaces of GI lumen, lungs, etc.) property of nanocrystals has been studied by various research groups through adsorption studies either under flow, or at static conditions and desorption studies.

Adsorption studies under flow were performed by observing the in vitro adsorption of micron sized particles (2–3 μ) from a flowing liquid film of dilute suspension onto the surface of rat intestinal strips. The steady state fraction of adsorbed particles was found to increase with the length of the intestinal strip and decrease with the flow-rate. For adsorption, the negatively-charged particles had to traverse a potential energy barrier of inter-particle electrical repulsion and also the negatively-charged mucous.

**Figure 3:**
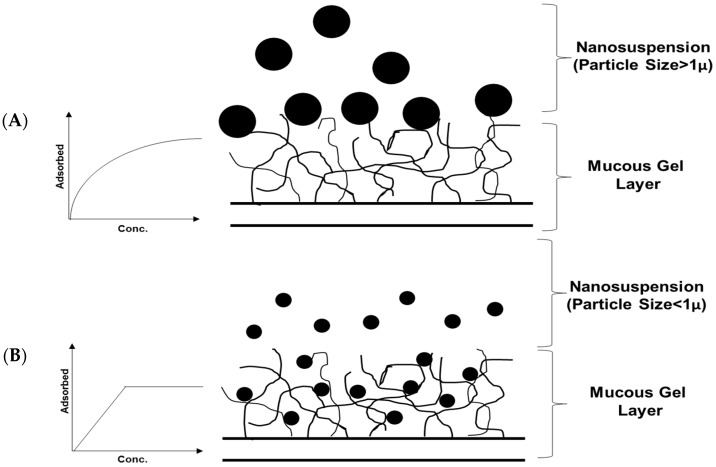
Adsorption Isotherms in case of (**A**) Particle Size Greater than 1 μ; (**B**) Particle Size Lesser than 1 μ.

Besides this, performing kinetics and adsorption isotherms is another approach to study adhesion. Interaction under static conditions was determined by studying adsorption of latexes to rat intestinal mucosa. It was proposed that the kinetics of adsorption was size dependent. In view of particle size, the mucous layer forms a porous rather than a smooth adsorbent. Isotherms of small latexes (230–670 nm polystyrene latexes) displayed characteristic adsorption isotherm of adsorbates, which penetrate into the porous adsorbent. Herein, the linear increase in the isotherm indicates the creation of new adsorption sites when the bulk particle concentration rises. These sites are accessible for further adsorption upto the saturation of the available sites indicated by an isotherm plateau. On the other hand, larger particles adsorb as a Langmuirian type. The adsorbate adsorbs in a monolayer on the surface of the adsorbent, which behaves like a smooth surface (Refer to Figure 3) [9,10].

### 2.4. Improved Permeability

Nanocrystals in the size range of 200–300 nm offer the advantages of enhanced permeability across the skin and mucosal membranes. Transdermal delivery is feasible only for a few drug molecules. This is mainly owing to the constraint in permeation of clinically effective concentrations of drugs through the skin barrier. This constraint is more marked in case of poorly water soluble drugs. Inspite of the enhanced permeation properties of water insoluble drugs due to its lipophilicity, rate of drug release is the rate limiting step for these drugs. Enhanced permeability of drug nanocrystals is a size dependent property; wherein particles with size less than 40 nm were found to permeate the skin via the follicular route while larger size particles showed limited permeation due to the tight network of epidermal Langerhan’s cells. Another study reveals that when the particle size was higher than 5 μ, almost negligible permeation was seen through the stratum corneum, while particles in the range of 500–750 nm manifested better permeation into the hair follicle of the human skin [9].

### 2.5. Long-Term Stability

Nanosuspensions are endowed with long-term stability, which is also one of the requirements for an approved formulation product. The marked physical stability of nanosuspensions is mainly owing to dearth of aggregation and absence of “Ostwald ripening” phenomenon. This is achieved by incorporation of a suitable stabilizer. Nanoparticles dispersed in a medium tend to aggregate. The physical instability of the system is born out of the penchant of the nanoparticles to reduce the high surface energy created by the large interface between the solid and the medium [11]. Physical stability may be obtained by using either of the multiple kind of stabilizers, such as the ionic surfactants, non-ionic surfactants, and amphiphilic copolymers [12]. During the homogenization progress, the surfactant diffuses rapidly and covers the surface of the crystals, stabilizing the system by ensuring an electrostatic and static barrier between the crystals [13]. To provide sufficient stabilization of the nanocrystals, the surfactants employed should meet the following criteria; firstly, the stabilizer should have a sufficient affinity for the drug particle surface to stabilize the nanocrystals [14]. For example, the ionic surfactants such as sodium dodecyl sulfate (SDS) and lecithin could migrate to the solid–liquid interface and provide an electrostatic boundary thereby thwarting agglomeration of the particles. The non-ionic surfactants, such as Pluronic F68 and Birj 78, have multiple linkages of hydrophobic domains at their surface which could adequately interact with the hydrophobic functional groups of the compounds and ensure stabilization of the system by imparting a steric barrier between the particles [15]. Secondly, the surfactant should have a sufficiently high diffusion velocity to envelop the generated surface rapidly since homogenization is a very rapid process. Lastly, the amount of the stabilizer should be sufficient for complete coverage on the particle surface to accord sufficient electronic or steric repulsion between the particles. However, it not true that more the stabilizer, the better the tendency to form micelles containing a small number of dissolved drug molecules [16]. Hence, screening for an ideal type and amount of surfactant is very crucial for the product quality.

Literature reports state that a binary or ternary mixture of electrostatic and static stabilizer usually yields better stabilization. At present the screening of the surfactant/stabilizers is mainly based on previous literature reports and experiences or on the basis of solubility studies and contact angle measurements of the drug. The fundamental principle that must be observed to choose a stabilizer is that it must be acceptable and safe to the body. For the nanosuspensions to be administrated intravenously, the stabilizers should be screened in a more stringent manner. In addition, the choice of the stabilizers is limited by the formulation process, e.g., the surfactant which is in the liquid state (e.g., Tween 80) cannot be used if the nanosuspensions need to be converted into a dried powder. The lack of Ostwald ripening phenomenon is primarily owing to the narrow size distribution of nanosuspensions [17]. It was reported that the particles in the highly dispersed systems tend to grow, which is caused by the differences in saturation solubility in the milieu of variably sized particles. The phenomenon is referred to as Ostwald ripening [18]. The solute concentration is higher in the region of the smaller particles in contrast to that of the larger particles due to the greater saturation solubility of the smaller ones. Therefore, the molecules will diffuse from the area of the small particles to the area of the large particles motivated by the concentration gradient and recrystallization on the surface of the larger particles. The perpetual dissolution of the small particles and recrystallization on the surface of the larger ones usher the formation of microparticles.

A narrow size distribution of the nanosuspensions created by adequate homogenization process circumvents the disparity in the saturation solubility between particles of different size ranges and hence facilitates the absence of the Ostwald ripening and a long-term stability. Nanosuspensions also ensure adequate chemical stability, both in vitro and in vivo for chemically labile drugs, which otherwise have a short half-life. The surface layer of the drug nanocrystals offer protection to the drug beneath the surface against degradation. An ideal example of this is paclitaxel, a water-sensitive drug [18]. Paclitaxel undergoes degradation after 48 h even when placed at room temperature conditions. However, when formulated as a nanocrystal stabilized with poloxamer 188, the drug shows excellent stability over a period of 4 years.

## 3. Characterization of Nanocrystals

For the successful fabrication of a nanocrystal formulation, besides selection of the appropriate excipients, equally important is the characterization of the formulation to ensure that the necessary parameters responsible for the performance of nanocrystals are within the specified limits. The following sections discuss in detail the various characterization tests for the evaluation of nanocrystals.

### 3.1. Solid State Properties

The solid state properties (polymorphic crystal form, solvate (especially hydrate) form, degree of crystallinity) influences the apparent solubility and thereby the dissolution rate. Hence, it is crucial to determine these characteristics in nanocrystals. Ideally, thermodynamically most stable crystalline form is desirable to prevent the peril of solid state transformations during production, storage and/or administration. To increase the dissolution and bioavailability of nanocrystals, it is preferable to formulate the nanocrystals in a metastable crystalline form or even prepare the amorphous equivalent of nanocrystals. However, this is not being practiced commonly.

Different nanocrystal manufacturing conditions and procedures can have an impact on the ensuing solid state form. Furthermore, the environmental conditions affect the thermodynamically stable polymorphic form. For e.g., hydrate forms are generally more stable (and therefore less soluble) in aqueous media. Therefore if the drug is susceptible to hydrate formation, then the potential or factors triggering the said conversion should be extensively investigated during stability studies in different conditions [19]. X-ray powder diffraction (XRD), thermal analytical techniques (differential scanning calorimetry, thermogravimetry, etc.) and vibrational spectroscopy (infrared and Raman) are the most commonly used methods to determine and monitor the solid state form of nanocrystals.

### 3.2. Thermal Analysis

Differential scanning calorimetry (DSC) is one recurrently used method for studying the thermal behavior of drug and drug nanocrystals. The DSC studies are performed to check the status of crystallinity of drug and interaction of excipients and drug after production of nanocrystals. This is especially important for drugs occurring in different polymorphic forms. Moreover, certain top-down techniques like the high pressure homogenization can lead to particles with an amorphous fraction, thus leading to enhancement of saturation solubility. The DSC of pure drug, physical mixture of drug and excipients (stabilizer) and final formulation which may be in dried form is done. DSC can be classified based on the mechanism of operation, into two classes; heat flux DSC and power compensated DSC. In heat flux DSC, two pans are placed on a thermoelectric disk surrounded by a furnace containing sample and empty reference pan. The furnace is heated at a linear heating rate, and the heat is transferred to the sample and reference pan through the thermoelectric disk [19,20,21]. However, owing to the heat capacity (*C*p) of the sample, there would be a discrepancy in the temperature between the sample and reference pans, which is measured by area thermocouples, and the consequent heat flow is determined by the thermal equivalent of Ohm’s law: *q* = ∆*T*/*R* where *q* is “sample heat flow”, *T* is “temperature difference between sample and reference”, and *R* is “resistance of thermoelectric disk” [20]. In a power-compensated DSC, the sample and reference pans are placed in separate furnaces heated by separate heaters [19,21]. The sample and reference pans are maintained at the same temperature, and the difference in thermal power required to maintain them at the same temperature is determined and plotted against temperature or time. Kocbek et al. prepared nanosuspension of ibuprofen using Poloxamer 188 as a stabilizer. A single exothermic peak at an onset temperature of 74.8 °C was seen in the DSC curve of pure ibuprofen, due to its melting. The DSC curve of Poloxamer 188 also manifests a single endothermic peak with an onset temperature of 51.4 °C. Two distinct endothermic changes in the DSC curve of freeze-dried ibuprofen-Poloxamer 188 nanosuspension were observed. The first endothermic change appears as a tall narrow peak with an onset temperature at 39.4 °C and the second as low broad peak with temperature of maximum at 56.8 °C, where it was not possible to analyze the onset temperature. These results indicated the formation of a eutectic mixture of the drug and Poloxamer 188. In this case, the peak at the lower temperature represents the melting of the eutectic system, and the second, change at the higher temperature, represents the melting of the excess component. Based on the position of the second peak, it can be estimated that ibuprofen is the surplus component left after the eutectic has been formed. Therefore the increased dissolution rate of the drug can be explained by formation of the eutectic system and submicron sized drug crystals produced during the formulation of nanosuspensions [11].

Among other thermal techniques, hot stage microscopy (also known as Thermal Microscopy or Thermomicroscopy) is a combination of microscopy and thermal analysis to enable the study and physical characterization of materials as a function of temperature and time. Hot stage microscopy not only aids in screening and characterization of polymorphs, but also identification of crystalline and amorphous region of nanocrystals. Yin et al. developed nanocrystals of a new drug moiety BMS-347070 by spray-drying with a surfactant Pluronic F127. The authors used hot-stage microscopy to compare the drug processed with Pluronic F127 and micronised pure drug. The hot stage microscopy images of the co-processed and micronized drug were collated at 100× magnification. Both the samples were put on slide in the same field of view. The slide was heated to 250 °C at a rate of 10 °C/min. The drug particles remaining in the molten Pluronic were found to be much smaller compared to the pure drug, the size of which cannot be determined by optical microscopy [22].

Thermal analysis may also be performed by thermogravimetric measurements or differential thermal analysis (DTA). Huang et al. used DTA for the thermal analysis of SKLB610 nanosuspensions at a heating rate of 10 °C/min in the range of 25–600 °C. The DTA curve of SKLB610 displayed a drug melting peak at 155.7 °C. No such peak was observed with the nanoparticles. Pure SKLB610 was also linked with another peak at 132–133 °C. The melting peak of SKLB610 in nanosuspension was relatively blunt as compared to that of pure SKLB610, apparently due to the preparation process. Amorphous domains were also generated on the particle surface [23].

### 3.3. X-ray Diffraction (XRD)

X-ray diffraction studies are usually performed for the confirmation of drug crystallinity following its conversion to a nanocrystal formulation. When X-rays interact with a crystalline substance, a diffraction pattern is obtained. Every crystalline substance gives a specific pattern; the same substance always yields the same pattern; and in a mixture of substances each produces its pattern independently of the others. The X-ray diffraction pattern of a substance therefore represents the unique fingerprint of the substance. Koneti et al. compared the top-down and bottom-up approaches for the preparation of nanosuspensions of glipizide based on their XRD output. The authors carried out XRD to analyze the modification in the crystalline nature of the drug following its conversion into nanocrystals. The diffraction pattern of pure glipizide powder manifested several sharp high intensity peaks at multiple diffraction angles (2θ values) signifying that the drug existed as a crystalline material. Glipizide nanosuspension displayed peaks almost similar in intensity and position, indicating that the crystallinity of the drug is intact when the nanosuspension is produced by liquid antisolvent precipitation method. In addition, the powder XRD study of spray dried nanosuspension prepared by top-down process (high speed milling) showed negligible shift in the main peaks as compared to pure drug. The characteristic peaks for milled and unmilled drug were observed at the same 2θ values. A slight decrease in intensity of peaks was observed with spray dried nanosuspension operated at higher milling speed [24].

### 3.4. FT-IR Studies

Chemical properties of drug and interaction with excipients are evaluated by FT-IR studies. Liandong et al. formulated and evaluated curcumin nanocrystals for pulmonary delivery. FTIR studies of the pure drug and the developed dry powder inhalation (wet-milling followed by spray-drying) were done for evaluation of change in chemical properties of the drug. Based on the position IR peaks in the formulation compared to that of the pure drug, it was concluded that milling and spray drying did not change the chemical composition of curcumin [25].

### 3.5. Raman Spectroscopy

Raman spectroscopy is a spectroscopic technique based on inelastic scattering of monochromatic light, originating from a laser source. Inelastic scattering means that the frequency of photons in monochromatic light amends following interaction with a sample. Photons of the laser light are absorbed by the sample and then reemitted. Frequency of the reemitted photons is shifted up or down compared to that from the original monochromatic frequency. This phenomenon is referred to as the “Raman Effect”. This shift provides information about vibrational, rotational and other low frequency transitions in molecules.

Waard and colleagues developed a novel bottom-up process to fabricate drug nanocrystals termed as “controlled crystallization during freeze drying” (CCDF), wherein a solution of an organic solution of a poorly water-soluble drug and an aqueous solution of a matrix material are mixed and freeze dried at specific conditions to allow the drug and the matrix to crystallize. The size of nanocrystals in this process was influenced by factors such as the freezing rate. Hence, to determine during what stage of the process the solutes crystallized and how the freezing rate impacted the particle size, the crystallization process was monitored by Raman Spectroscopy. The sample to be studied comprised of the drug (Fenofibrate; FNB), the solvent (tert-butyl alcohol; TBA), mannitol and water. The Raman probe was placed directly above the sample in the freeze dryer and individual stages of the CCDF process were separated and prolonged to allow measurement of the complete phase changes. CCDF comprises of three consecutive steps: freezing, increasing the temperature, and drying. The in-line Raman measurements showed that the first two steps, the freezing step and the crystallization step are critical steps that determine the final size of the fenofibrate crystals [26].

As mentioned earlier, the solid state characteristics of nanocrystals are influenced by the production method. With bottom-up techniques partial amorphousness is commonly manifested, with pernicious effects on the stability of the nanocrystals. Liquid atomization based techniques, like spray drying or electrospraying, are markedly susceptible to generating a final product in the amorphous form (partially or fully). However, full crystallinity can be obtained after production by annealing. The high shear stresses associated with wet media milling and high pressure homogenization may also result in polymorphic changes. However, if the process is performed in an aqueous environment, water serves as a plasticizer (raising molecular mobility) and reduces the propensity for sustained formation of amorphous material. Ali et al. prepared hydrocortisone nanosuspensions by both wet-milling and microfluidic nanoprecipitation [27]. With both methods, the particle sizes were approximately 300 nm, yet the product obtained via milling was crystalline, while precipitation resulted in a predominantly amorphous product. In vivo tests with rabbits following ocular delivery, demonstrated comparable bioavailability with both the formulations and when compared against the drug solution, the bioavailability was found to be almost two-fold. However, the differences were evident in stability tests, the crystalline wet-milled nanosuspension was stable for two months (unrevised particle size), but the particle size of the amorphous precipitated nanosuspension had increased to 440 nm. Lai et al. formulated piroxicam nanocrystals with poloxamer 188 as a stabilizer by high pressure homogenization [28]. While the raw material was predominantly form I, a mixture of monohydrate and form III composed the formulated nanocrystals. The solubility of form I is 14.3 mg/L, while that of form III is 17.0 mg/L. In this case, the solubility was increased not only due to the smaller particle size, but also due to the formation of the higher energy solid-state forms. Pireddu et al. studied two different diclofenac sodium crystal forms for transdermal drug delivery [29]. Nanocrystals were produced by wet ball milling, with poloxamer 188 used as a stabilizer. There were no significant differences between the particle size of the two polymorphs when the same milling protocol was used, but differences in the stability with respect to the particle size were seen during the 90 days of stability testing. The milling did not alter the polymorphic form of the drug. The crystallite size of the milled polymorphs was calculated based on XRPD peak width broadening. It was observed that for polymorph 1, the crystallite size was around 90 nm while for polymorph 2 it was around 65 nm. In vitro penetration and permeation studies revealed that all the nanosuspension formulations demonstrated an improved drug penetration compared to the commercial gel formulation. Interestingly, though the two polymorphic forms varied in their permeation properties; when administered as coarse suspensions, their nanosuspensions behaved similarly.

### 3.6. Particle Size and Size Distribution

Size and size distribution are important characterizations of the nanosuspensions because they direct the other properties, such as physical stability, saturation solubility and dissolution velocity, and even clinical efficacy. The smaller the particle size, the higher the surface energy of the particles, which promotes aggregation. The most frequently used techniques for particle size measurements of nanosized systems are dynamic light scattering techniques, static light scattering techniques and microscopy. The mean particle size of nanosuspensions is typically analyzed by dynamic light scattering also known as photon correlation spectroscopy (PCS) [30]. It has advantages of yielding accurate results and fast and easy measurement. However, this technique is not feasible to analyze particles larger than 6 µ. Apart from the mean particle diameter, PCS can also yield the width of the particle size distribution, referred to as the “polydispersity index” (PI). The PI value ranges from 0 (monodisperse particles) to 0.500 (broad distribution), and is a crucial index that governs the physical stability. For a long-term stability the PI should be as low as possible.

Techniques for the detection of larger particles are optical microscopy and low angle static light scattering (laser light diffraction), especially for the nanosuspensions that are meant for parenteral and pulmonary delivery. The advantage of light microscopy is the visible and therefore yields a doubt free result, however, a major drawback is lack of any statistical significance because it is not possible or very time consuming to analyze 10,000 particles or more, necessary for a valid analysis. Laser diffractometry (LD) is a robust technique and has the advantage over all the other techniques to be able to analyze large particles, small nanoparticles and mixtures of small and large particles within only one single measurement. The LD yields a volume distribution and possesses a measuring range of approximately 0.05–80 µm up to a maximum of 2000 µm, depending on the type of equipment employed. Typical characterization parameters of LD are diameters 50%, 90%, 99%, represented by D_50_, D_90_, and D_99_, respectively (i.e., the D_50_ implies that 50% of the volume of the particles is below the given size). The disadvantages of laser diffraction techniques rose with the need of analyzing nanoparticles with a technique being originally intended for the measurement of larger particles in the micron range. Since, laser diffraction is a simple and rapid method it was aimed to extend the measuring range (e.g., from 400 nm to 2000 m) to a broader range, being able to analyze even very small particles (e.g., from 20 nm to 2000 m). However, practically it is only possible to analyze particles from about 400 nm and larger using this technique [30].

Laser diffraction is not feasible for measurement of particles smaller than about 400 nm, because the intensity of diffracted light decreases with decreasing size. However, modern LD instruments can analyze particles starting from 20 nm up to those in micron range or even larger. The appendage of the measuring range for very small particles was possible by introducing a second, supplementary technique, into the measurement. The additional technique gains more information about the particles by determining other optical phenomena (e.g., scattering intensities in different directions) [31,32]. Thus, the additional techniques are different to pure laser light diffraction. The additional information from the supplementary technique is then amalgamated into the size analysis of the LD measurement, which leads to a pooled result of pure LD and supplementary technique. Therefore, strictly spoken, today’s LD measurements are not only pure LD measurements, but an integrated report of two different techniques. However, the additional technique may also overestimate the presence of the nanoparticles by overlooking larger particles (e.g., large crystals and/or aggregates/agglomerates) [33]. This finding is of extraordinary importance, because most often LD is used to detect possible large particles besides a nanosized major bulk population, or to evidence the absence of such large crystals, which is not possible in PCS measurements. Hence to conclude, particle sizing of nanocrystal formulations will only lead to meaningful results if all the aforementioned parameters are considered. Moreover, it should be noted that the particle size data of a nanosuspension obtained by LD and PCS are dissimilar, because the LD data are volume based and the PCS mean particle size is a light-intensity weighted size.

A Coulter counter analysis is essential for nanosuspensions to be administrated intravenously. In contrast to the volume distribution of the LD analysis, the Coulter counter data give an absolute value that is the absolute number of particles per volume unit for the different size classes. The size of the smallest blood capillary is about 5 µm, so even a small content of particles greater than 5 µm may cause capillary blockade or emboli formation. Hence the content of microparticles in nanosuspensions should be controlled strictly by Coulter counter analysis [34].

Obstacles in particle size analysis not only arise from the instrumental setup alone, but also from the material to be analyzed. General concept states that the stability of the sample during analysis is the most important prerequisite for correct and reproducible results [35]. However this is not always easy to achieve and sometimes changes are not even deciphered. Possible alterations or instabilities of a sample are for instance agglomeration or dissolution. Therefore the analysis of samples with high solubility and/or increased dissolution velocity might be especially sensitive to such changes.

Apart from these, the other techniques used for the particle size analysis include confocal laser scanning microscopy, scanning probe microscopy, and scanning tunnelling microscopy.

### 3.7. Particle Shape and Morphology

Ideally, the shape or morphology of the nanocrystals can be determined using a transmission electron microscope (TEM) and/or a scanning electron microscope (SEM). A wet sample of suitable concentration is needed for the TEM analysis. When the formulated nanosuspensions are to be converted into a dried powder (e.g., by spray drying or lyophilization), a SEM analysis is crucial to monitor alterations in the particle shape and size before and following the process of the water removal. Principally, agglomeration may be observed following water removal, leading to an increase in the particles size. Such changes and more can be observed through SEM. To play down the magnitude of the increase of particle size, some excipients are included as “protectants”. Mannitol is usually used as a cryoprotectant in lyophilization, which recrystallizes around the nanocrystals during the water-removal operation, thus preventing particle interaction and agglomeration. Agglomeration within a certain limit is allowable when the final particle size is still in an acceptable range. Moreover, the dried powder should be easily redispersed into stable nanosuspensions. The shape of the drug crystals depends on their crystalline structure [34,35].

Atomic Force Microscopy (AFM), a kind of scanning probe microscope is designed to measure local properties, such as height, friction, magnetism with a probe. AFM was used to investigate the morphology and surface properties of probucol nanocrystals following dispersion of probucol/polyvinylpyrrolidone (PVP)/sodium dodecyl sulfate (SDS) ternary ground mixture into water. The observed particles had core-shell structure, i.e., drug nanocrystals enveloped in a PVP and SDS complex. The AFM phase image and the force curve analyses indicated that probucol nanoparticles with PVP K17 manifested layer structure, compared to those with PVPK12. The structural difference was accountable in terms of the molecular states of PVP-SDS complex on the particle surface. These findings support not only the mechanism of drug nanoparticle formation but also the in vivo absorption results with the almost same particle size of 40 nm [36].

Surface plasmon resonance (SPR) analysis has been employed in interaction studies between solid drug surfaces and aqueous stabilizer solutions. Five structurally different PPO/PEO block co-polymers were used as stabilizers for indomethacin nanocrystals, and affinities between the stabilizers and solid drug surfaces were determined by SPR and contact angle measurements. Both techniques displayed a similar level of efficiency of binding to the solid surfaces [37]. The interaction measurements were corelated to successful formulation of nanocrystals with the same drug-stabilizer systems by wet ball milling. Hence, it was concluded that merely the interaction forces cannot determine the most efficient stabilizer, but moderate affinity with longer PEO chains, which are efficient for steric stabilization, formed best nanosuspensions.

Particle shape is of prime importance when the nanocrystals are to be formulated as dry powder inhalers (DPIs) for direct lung delivery of the drugs. Aerodynamic diameter is a critical parameter that determines the lung deposition of the DPIs. Different particle shapes yield different drag forces and particle terminal settling velocities, which in turn influence the lung deposition of the DPIs. Enhancing particle surface roughness would ascribe to decreased aerodynamic diameter of the particles. This would inturn have a higher possibility of deep lung deposition as compared to spherical particles. In another study, elongated particles were found to have better aerodynamic behavior as compared to spherical ones. Particle aerosolization is another factor responsible for deep lung delivery. Aerosolization is mainly influenced by the interaction between the particles and between the particle and the wall of the inhaler. Particle interactions are linked to the van der Waals forces, which are the particle surface morphology, size, shape, electrostatic properties and hygroscopicity. Particle shape that possess low contact area and van der Waals force have a lower tendency to aggregate and hence can be readily dispersed in the air. Elongated particles are not ideal for aerosolization owing to their large attractive forces. A study by Hassan et al. evaluated the flowability, aerosolization, and deposition properties of particles of different shapes like sphere, cube, needle, and pollen [38]. Pollen shaped particles are found to display better flowability, aerosolization, and deposition properties compared with other particle shapes.

### 3.8. Particle Surface Charge

The surface charge of the particles is one of the factors influencing the physical stability of nanosuspensions. The higher the particles are equally charged, greater is the electrostatic repulsion between the particles and greater is the physical stability. The particle surface charge is ideally quantified in terms of the “zeta potential”, which is measured via the electrophoretic mobility of the particles in an electric field. The particle charge can be measured in surface charge per unit, determined by colloid titration.

In general particles inherently possess a surface charge, owing to the dissociation of surface functional groups, referred to as the Nernst potential. The degree of dissociation of the functional groups depends of the pH of the suspension, therefore zeta potential is a pH dependent characteristic.

In an electrolyte containing media, ions from the dispersion medium adsorb onto the particle surface. For this model description a negative Nernst potential is assumed. In general the first absorbed monolayer of ions comprises of negatively charged, fixed and dehydrated ions, termed as the Helmholtz layer. The second monolayer absorbed consists of positively charged, fixed but hydrated ions, referred to as the outer Helmholtz layer. Both Helmholtz layers together are designated as the Stern layer. The uncompensated negative charge of the surface is compensated by freely diffusing counter ions in the so called “diffuse layer”. The border of the diffuse layer is defined where the particle surface charge is fully compensated. The zeta potential is determined by measuring the electrophoretic particle velocity in an electrical field. During the particle movement the diffuse layer is shed off, hence the particle acquires a charge due to the loss of the counter ions in the diffuse layer. This charge at the border of the shedding is termed as the zeta potential.

The measurement itself is a particle electrophoresis, the particle velocity is determined via the doppler shift of the laser light scattered by the moving particles. The field strength applied is generaly 20 V/cm. The electrophoretic mobility was converted to the zeta potential in mV using the Helmholtz–Smoluchowski equation. At standard measuring conditions (room temperature of 25 °C, water) this equation can be simplified to the multiplication of the measured electrophoretic mobility (µm/cm per V/cm) by a factor of 12.8, yielding the ZP in mV.

The measurement of the zeta potential allows the prediction about the storage stability of submicron colloidal dispersion [35,39]. In general, particle aggregation is less likely to occur if particles possess enough zeta potential providing sufficient electric repulsion, or enough steric barrier providing sufficient steric repulsion between each other. According to the literature, a zeta potential of at least −30 mV for electrostatic and −20 mV for sterically stabilized systems is desired to obtain a physically stable nanocrystal suspensions [18]. The upper limit for the zeta potential is +30 mV.

### 3.9. Dissolution of Nanocrystals: Apparent Solubility and Supersaturated State

Thermodynamic solubility implies the solubility of the most stable crystalline form of the drug in a given medium at a specified pressure and temperature. Solubility can temporarily be higher than the thermodynamic solubility. This may be observed with amorphous forms, metastable polymorphic forms, or nanosized drug particles. This enhanced solubility has been designated with diverse terms, such as kinetic or apparent solubility. Since apparent solubility of nanosized particles is higher than the thermodynamic solubility of the material, dissolution of nanocrystalline material is likely to lead to a supersaturated solution. This is termed as the “spring effect”. Ige et al. studied the saturation solubility of fenofibrate nanocrystals, which had been reduced in size from 80 µm (bulk drug) to 460 nm (nanocrystals). The thermodynamic solubility of the bulk drug in aqueous 0.5% and 1% sodium dodecyl sulfate solution, was 6.02 and 23.54 µg/mL, respectively, while the corresponding values for drug nanocrystals were 67.51 and 107 µg/mL, respectively [40].

In another study, the intrinsic dissolution rates and surface concentrations of indomethacin nanocrystals with two different poloxamer stabilizers were studied [41]. Intrinsic dissolution rates were measured with a channel flow system. The intrinsic dissolution rates were profoundly influenced by the particle size and the stabilizer. With the smallest nanocrystals (580 nm), the intrinsic dissolution rate with poloxamer F68 as a stabilizer was 0.50 µg/min/mm^2^, while that for poloxamer F127 was 0.31 µg/min/mm^2^. The dissolution rate of bulk indomethacin was also determined, and found to be considerably lower at 0.05 µg/min/mm^2^. Surface concentrations have also been measured with UV-imaging. Herein again, differences in concentrations were affected by both particle size and stabilizer. For e.g., for nanocrystals of a uniform size (580 nm), the surface concentration after 10 min of dissolution was 28.7 mg/L for Pluronic F68 while with Pluronic F127 the concentration was 22.1 mg/L. The surface concentration with bulk indomethacin was only 2.1 mg/L.

Liu with coworkers studied the effect of ultracentrifugation and filtration on the dissolution results. Indomethacin, the model drug used was found to interact with both the type of filter tested as well as the centrifuge tube material. Undissolved drug particles in the sample can be recognized employing multiple wavelength for the analysis. Sarnes with colleagues determined drug concentrations in solubility testing of nanocrystalline samples with UV-spectrophotometer. The drug concentration determinations were performed at wavelength equivalent to the absorption maximum of the drugs; while the absence of undissolved particles was confirmed with the wavelength at which the absorbance of the excipient and drug was negligible [37].

Eventually, precipitation may sooner or later occur until the concentration is equivalent to the thermodynamic solubility. Furthermore, changes in the composition and pH of the solution such as in the gastrointestinal tract will affect solubility and hence the tendency for crystallization. Therefore, the supersaturated state should be maintained and precipitation hindered to enhance in vivo bioavailability. Some polymers, such as polyvinyl pyrrolidone (PVP), methacrylate co-polymers, hydroxypropyl methylcellulose (HPMC), and hydroxypropyl methylcellulose acetate succinate (HPMC-AS) are effective at maintaining (or at least helping to maintain) supersaturation. This is known as the “Parachute effect”. Solid dispersions, especially where the amorphous drug is dispersed on a molecular level within the polymeric crystallization inhibitor, are well established as parachute promoters. However, the permeation from supersaturated solutions may be thwarted by the precipitation inhibitor, as is the case often with solubilizing agents, when the drug favors the formation of micelles instead of permeation [41,42].

The parachute effect of the polymer can be due to a combination of mechanisms. First, the polymers can themselves increase the thermodynamic solubility of the drug (also termed as the co-solvency effect), which lowers supersaturation and consequently the thermodynamic driving force for crystallization (this also leads to an additional spring effect with the polymer). Through drug-polymer interaction in solution via electrostatic bonds, van der Waals’ forces or hydrogen bonding, even the addition of small amounts of polymers such as PVP and HPMC to solution can significantly increase the aqueous solubility. Second, polymers adsorbed on solid surfaces (e.g., with nanocrystals) can block the interaction of already dissolved drug molecules with crystal surfaces and thereby crystal growth. Electrostatic bonds, van der Waals’ forces or hydrogen bonding can all influence the interaction between the polymer and crystal faces, and therefore the degree of crystal growth inhibition. Moreover, the viscosity of the polymer solution may also inhibit the diffusion of the molecules, which limits crystal growth [43].

Ghosh et al. formulated nanocrystals from a poorly soluble drug with TPGS or TPGS with a co-stabilizer (HPMC, PVP, poloxamers). In vivo studies with dogs revealed a 9 times higher AUC value and 5 times higher *C*_max_ values for the nanosuspension against the coarse drug formulation. The physical stability during storage with TPGS alone was considerably lower than for the mixed systems [44].

Ueda et al. studied the maintenance of supersaturation with amorphous and nanocrystalline formulations of carbamazepine. The phenomenon of supersaturation was further studied by conducting real-time monitoring with NMR spectroscopy of the dissolved drug with both amorphous and nanocrystalline drug in supersaturated solution. Based on ^1^H-NMR measurements, the dissolved concentrations for nanocrystalline carbamazepine were nearly constant for 50 h. Based on this, the authors concluded that nanocrystal formation lowered the degree of supersaturation, leading to a relatively stable supersaturated solution of carbamazepine. The presence of nanoparticles also suppressed the formation of large precipitates. For spray dried amorphous carbamazepine, the initial concentration was higher but it then dropped below the concentration of the nanocrystalline sample, demonstrating that the higher supersaturation was more kinetically unstable with fast precipitation/crystallization of large microparticles. The particle size of nanocrystals in this study was approximately 150 nm [45].

### 3.10. Permeation Study

Nanocrystal based drug delivery could be very effective for improving dermal bioavailability of drugs with poor solubility. Indeed, in addition to increased saturation solubility and dissolution rate, nanocrystal also exhibits the property of increased adhesiveness to the skin thus facilitating the dermal delivery [46,47]. The two mechanisms by which drug is delivered to the skin; first one is simple increase of concentration gradient between formulation and skin and the second mechanism involves hair follicles. Nanocrystals with an appropriate size (approximately 700 nm) can deposit into these shunts, which act as a depot from which the drug can diffuse into the surrounding cells for extended release. The factors including the particle size of the drug crystals, surface properties of the carrier, drug-stabilizer interaction are to be considered while poorly soluble drug is formulated for dermal drug delivery. The nanocrystal based drug delivery to the eye can be exploited for improving retention and penetration of drug in to the eye. The possible mechanism for this is not only to increase solubility in lachrymal fluid but also to produce adhesive properties. Nanocrystals may be used not only to increase solubility in lachrymal fluids of poorly soluble drugs, but also to produce adhesive properties (determined by the nature of the surfactant in the formulation) that can be exploited for improving the retention and penetration of drugs into the eye. Nonionic surfactants are preferred over ionic because they are generally less irritating [48]. The permeation studies are usually done by using the Franz diffusion cell apparatus. The human cadaver skin, pig ear skin, rat skin, and pig skin can be used for the same. The nanosuspension is compared with the coarse suspension or the marketed one. The dermal absorption of formulation in vitro is done by application of the test substance in a suitable formulation to the surface of a skin sample, which is placed between the donor compartment and the receptor compartment of a diffusion cell. Two types of diffusion cells are available i.e., static or flow-through [49,50]. In Static diffusion cells, fresh perfusate is added after each sampling. Flow through cells use a pump to pass perfusate through the receptor chamber and collect flux by frequently collecting the perfusate. Static diffusion cells can be sub divided as horizontal or vertical on the basis of skin orientation. The majority of skin absorption studies are conducted using horizontal cells, with the skin surface open to the air. The inner material of Diffusion cells is inert non-adsorbing with receptor chamber volumes of about 0.5–10 mL and surface areas of exposed membranes of about 0.2–2 cm^2^ [51,52]. The test should be carried out with minimum of six skin samples. The receptor fluid, which must have an adequate ability to solubilize the test substance, is maintained in contact with under side of the skin from the time of application until the end of the collection of the receptor fluid. A very critical parameter is controlling the temperature of the receptor fluid throughout the experiment. The skin surface temperature in the diffusion cell should be kept to 32 ± 1 °C. The receptor fluid in static cells should be well stirred during the study.

### 3.11. Drug Absorption from Nanocrystalline Formulations

Drug absorption is directly related to both solubility and permeability and inversely related to lipophilicity. Dissolution from nanocrystals is followed by permeation of the dissolved drug across the gastrointestinal wall (in the same way as drug from a solution formulation). Besides increasing permeation of the drug due to elevated dissolved concentrations, stabilizers present in the formulation themselves interact with cells of the epithelial layers to promote permeation.

Li with colleagues studied the effect of drug physicochemical properties on oral bioavailability [53]. They studied five different drugs and nanocrystals were formulated using the same stabilizer, poloxamer 188, by high pressure homogenization. Particle size obtained for all the tested drugs was almost 430 ± 30 nm. The AUC values in all the cases was 1.4–7.2 times higher as compared to drug microsuspensions following oral administration to rats. Melting point, log *p* value and polar surface area were found to have an influence on drug absorption. Drugs with low melting point, log *p* value approximately 5 and polar surface area value between 50 and 60 manifested higher absorption with the same sized nanocrystals.

Many stabilizers used for nanocrystal products (vitamin E TPGS, poloxamers, polysorbates) are also P-gp inhibitors [54]. PEG chain length (between 200 and 6000 Da) in TPGS may influence the inhibitory activity and the best inhibition is observed with PEG chain lengths of 1100–1500 Da. In some cases, nanocrystals can be taken up by cells. This may be desirable (e.g., with cancer cell targeting) or undesirable (unpredictable pharmacokinetic profiles) depending upon the intended purpose of the nanocrystals. The uptake may vary between cell types and their phagocytotic/endocytotic potential, and the properties of nanocrystals such as size, morphology, stabilizer type, and surface charge. The wide array of applications of nanocrystals, and their potential importance highlights the need, in some cases, for a thorough comprehension of the nanocrystal behavior on the cellular and tissue levels. Chen and Li [55] studied the mechanism of cellular uptake of paclitaxel nanocrystals. They found out that nanocrystals were internalized by KB cells at higher concentrations compared to the solubilized formulations. Based on temperature dependent internalization and confocal imaging, they concluded that drug nanocrystals were possible to be taken up as solid particles probably via endocytosis. However, the uptake was affected by the surface layer of the nanocrystals. Thus, nanocrystalline chemotherapeutic formulation can perhaps intracellularly form a lethal microenvironment for the cell when the drug nanocrystals are slowly dissolved inside the cells. This can be difficult to access for the solubilized drug. The mean particle size of nanocrystals in this study was from 250 ± 30 nm. The potential of TPGS stabilized paclitaxel nanocrystals to reverse P-glycoprotein drug-resistance in P-gp overexpressing H460 cancer cells was evaluated by Gao with colleagues [56]. It was found that TPGS as a stabilizer efficiently lowered drug resistance of the studied cells. It is known that due to the enhanced permeation and retention (EPR) effect, drug nanoparticles accumulate in the tumor tissues following an intravenous injection. However, therapeutic efficacy is limited by overexpressing MDR related proteins like P-gp in resistant tumors. Though nanosized materials can be taken up via endocytosis by cells, but following dissolution into cellular cytoplasm they can be pumped out by P-gp efflux system. Hence, utilization of simultaneous lowering of P-gp activity with endocytosis of nanoparticles can increase the therapeutic efficiency, like was the case with TPGS coated paclitaxel nanocrystals. Drug and P-gp inhibitor should be at the same time inside the cells. This was not realized when TPGS was given in solution together with free paclitaxel molecules, but with TPGS coated paclitaxel nanocrystals it worked. While electron microscopy and fluorescence imaging are the two principle techniques to image the physical interaction of nanoparticles with cells, including their uptake and localization within the cells they have some drawbacks (e.g., lack of chemical specificity and inability to analyze live cells with electron microscopy), and complications with fluorescent labels, including label leaching and overestimation of nanocrystal internalization when the fluorescent labels but not necessarily the nanocrystals themselves enter the cells. Thus, it is worth considering novel analytical techniques in this context.

Confocal Raman microscopy and coherent anti-Stokes Raman scattering (CARS) microscopy are novel label-free, chemically specific and non-destructive methods with potential for label-free imaging of nanocrystal-cell interactions. With these techniques submicron particles may be analyzed provided they have a sufficiently strong Raman or CARS signal (the resolution and speed is better for the inherently confocal CARS technique, while chemical specificity is better for Raman microscopy) [57]. In a proof of concept study that also had clinical relevance, Darville et al. imaged the fate of nonfluorescent nano/micro crystals paliperidone palmitate, an antipsychotic prodrug in macrophage cell cultures and histological sections using CARS microscopy [58]. The commercially available product Xeplionr is a long-acting aqueous suspension for intramuscular injection with a measured median volume based equivalent sphere diameter of approximately 1000 nm.

The palmitate prodrug aids to reduce solubility and associated dissolution rate, thereby sustaining the release of paliperidone [59]. In vivo complex and variable pharmacokinetic profiles have been observed and in rats the formation of granulomatous tissue in the region of the intramuscular nanocrystals has been observed. The inflammatory response leads to particle agglomeration, phagocytosis and radial angiogenesis in the rats resulting in multiphasic systemic absorption of the paliperidone. CARS microscopy was used to investigate the fate of the paliperidone palmitate nanocrystals with macrophage cells in vitro and histological sections in situ. The nanocrystals were imaged in both fixed and live cells using the CH_2_ stretching resonance at 2845 cm^−1^, mainly associated with the palmitate moiety (the nanocrystals were resolved from endogenous lipid in this case through geometrical differences, and an otherwise weak lipid signal from the cells was used, although with other drugs a CARS resonance resolved from lipid signals could be used for chemical specificity). In tissue sections, intracellular nanocrystals were imaged within the granulomatous tissue.

## 4. Conclusions

Drug nanocrystals are a highly feasible option for enhancing the bioavailability of poorly water-soluble drugs. For efficient performance of the nanocrystals, evaluation of the performance characteristics is a critical part. Proper tools and methods for the evaluation of the nanocrystals have been developed and applied.
